# Are childhood diseases associated with Molar-Incisor Hypomineralization? – Results from an observational study

**DOI:** 10.1007/s00784-026-07182-2

**Published:** 2026-10-07

**Authors:** Elena Pernegger, Nour Ammar, Karl-Ferdinand Fresen, Ramy Gaballah, Helen Irina Schill, Stefanie Amend, Norbert Krämer, Jan Kühnisch

**Affiliations:** 1 Department of Conservative Dentistry, Periodontology and Digital Dentistry, LMU University Hospital, Ludwig-Maximilians-Universität München, Munich, Germany; 2 Department of Paediatric Dentistry and Dental Public Health, Faculty of Dentistry, Alexandria University, Alexandria, Egypt; 3 Department of Paediatric Dentistry, Medical Centre for Dentistry, University Medical Centre Giessen and Marburg, Campus Giessen, Giessen, Germany; 4 Klinikum der Ludwig-Maximilians-Universität München, Klinik für Zahnerhaltung, Parodontologie und digitale Zahnmedizin, Goethestraße 70, 80336 München, Germany

**Keywords:** Enamel Hypomineralization, Molar Hypomineralization, Paediatric Dentistry, Aetiology, Antibiotics

## Abstract

**Objective:**

This observational study aimed to investigate the potential association between reported childhood diseases and medications during early infancy and the later appearance of molar incisor hypomineralization (MIH).

**Materials and methods:**

As part of a prospectively designed epidemiological study, 5,418 Bavarian children aged 8 to 10 were examined for the presence of caries (WHO standard) and MIH (EAPD criteria). In addition, each child received a questionnaire to be completed by their parents. A total of 1,315 questionnaires were returned, containing complete information on childhood diseases, medication and socioeconomic status. Marginalized zero-inflated negative binomial regression models were constructed.

**Results:**

A total of 263 children (20.0%) were diagnosed with MIH, with a mean of 3.3 (± 2.0) MIH-affected teeth. In cases of bronchitis (IRR 1.25 with 95% CI 1.07–1.46), otitis media (aOR 1.91 with 95% CI 1.30–2.80), eye diseases, allergies, speech disorders, worm infections and cardiac diseases, significant associations were found for the presence of MIH. In addition, a significant association between antibiotic use and MIH was confirmed (first year of life: IRR 1.45 with 95% CI 1.15–1.83; aOR 1.86 with 95% CI 1.13–3.05/second year of life: IRR 1.35 with 95% CI 1.05–1.77; aOR 1.36 with 95% CI 1.05–1.77).

**Conclusions:**

The associations were mostly associated with low case numbers and accordingly cannot explain the documented MIH prevalence of 20.0%. Therefore, childhood diseases and antibiotic use probably do not solely contribute to MIH development.

**Clinical relevance:**

MIH aetiology remains unclear, indicating a need for further research.

## Introduction

Molar-incisor hypomineralization (MIH) is a developmental disorder of the first permanent molars and incisors that is likely to occur in infancy and is typically diagnosed at school age. A meta-analytic evaluation of globally available epidemiological data revealed an estimated MIH prevalence of approximately 15% [1–3]. Recent surveys from Germany reported a prevalence of 15.3% among 12-year-olds [4] and 17.5% among 8- to 10-year-olds [5]. Problematically, the aetiology of MIH is still unclear [6, 7] and is currently assumed to be multifactorial [8, 9]. Predisposing factors include perinatal factors [6, 10], such as bisphenol A exposure [11], antibiotic use in childhood [7, 12], genetic predispositions [9] and early childhood illnesses [2, 6, 7, 10, 12–14]. From a preventive perspective, only vitamin D has been documented to have a possible protective effect [15, 16].

When the influence of early childhood diseases on the development of MIH is examined in detail, positive associations have been demonstrated for a broad spectrum of illnesses in various studies. This was the case, for example, for the occurrence of fever [13, 17–19], respiratory diseases [13, 18–21], middle ear infections [17, 22–24] and asthma [13, 18–20, 25]. Positive associations between antibiotic use and the occurrence of MIH have also been reported [7, 12, 13, 22]. This is of clinical importance because antibiotic interventions are widely used as therapeutic approaches in the case of infections. Furthermore, little is known about correlations between analgetic use during early childhood and the later presence of MIH. Most recently published data suggest a positive association [26]. In view of the potential role of early childhood diseases and medications in the development of MIH, both should be considered as potential etiological variables.

With the aim of understanding the unknown aetiology of MIH, a recent cross-sectional epidemiological study [5] was supplemented with a retrospective questionnaire on the occurrence of diseases in early childhood. The aim of the present observational study was to correlate the prevalence and severity of MIH documented at the age of 8 to 10 years with the occurrence of childhood diseases, which were retrospectively recorded using a questionnaire. The null hypothesis to be tested was that there was no significant association between childhood diseases, antibiotic use, analgesic interventions and MIH.

## Materials and methods

This observational study was conducted between March and July 2023 in Bavaria, Germany, and included primary school children aged 8 to 10 years. The study concept was developed and carried out in collaboration between the Bavarian Working Group for Public Dental Health in Children and Adolescents (Bayerische Landesarbeitsgemeinschaft Zahngesundheit e.V., LAGZ), the Department of Paediatric Dentistry at Justus-Liebig-University in Giessen and the Department of Conservative Dentistry and Periodontology at Ludwig-Maximilians-University in Munich, in accordance with the applicable ethical standards of the Declaration of Helsinki [27] and approved by the Ethics Committee of the Medical Faculty at Justus-Liebig-University Giessen (project number 72/22). Before the study began, all children and their legal guardians at the randomly selected schools received written patient information detailing the study objectives, clinical procedures, questionnaire, data management and data protection regulations. Only children whose legal guardians provided written consent were included and examined. Participation in the study was voluntary at all times. This publication was prepared in accordance with the STROBE reporting guidelines for observational studies [28].

### Sample size calculation and study population

Based on population data from the Bavarian Statistics Office, the urban/rural distribution was set at a ratio of 25/75. In addition, the MIH prevalence was determined with a confidence interval of < 2.1%, which ultimately required a sample size of 5,000 individuals. Considering expected dropouts, 76 schools were initially selected at random. At the start of the study, however, it became apparent that the number of participating schools and children was lower than expected. It was, therefore, necessary to recruit an additional 40 schools. Ultimately, the number of participating children totalled 5,418 (mean age of 9.8 ± 0.7 years; 2,692 males and 2,726 females), who were examined at 87 Bavarian schools. Further information on the sample can be found in Fresen et al. [5].

### Clinical examination

All clinical examinations took place on the agreed-upon days in the children’s schools. The children were examined either on mobile patient couches or directly on school chairs in a separate room. The visual (tactile) examination included sterilized dental mirrors and blunt CPI probes (CP-11.5B6; Hu-Friedy, Chicago, IL, USA). To ensure adequate illumination of the oral cavity, the examiners used either halogen lamps (Haeberle Halux 50 S, 50 watts; Haeberle GmbH; Stuttgart, Germany) or their individual headlamps. In addition, it was possible to clean and dry all the tooth surfaces of each patient with cotton rolls and/or compressed air (portable compressor SNR MAC-A-002670; DTS Design, Mammendorf, Germany). In case of insufficient oral hygiene, the child was asked to brush their teeth carefully in the given school setting.

The clinical examination included the registration of the dental status, carious lesions and MIH-related developmental defects. The case report form allowed the documentation of multiple findings per surface. Caries was recorded using the dmft/DMFT index by the WHO [29]. Initial/ non-cavitated carious lesions (it/IT) were registered according to the ICDAS and UniViSS criteria [30–32]. Developmental defects in terms of MIH were diagnosed as demarcated opacities, enamel breakdowns, atypical restorations and extractions due to MIH according to the EAPD criteria [8, 33]. Hypomineralization with a diameter < 2 mm was not recorded [8, 33]. In general, repetitive documentation of the same dental defect in the clinical entities of caries and MIH was not allowed. Individuals with other developmental defects, e.g., hypoplasia, diffuse opacities/fluorosis or rare dental diseases, were consequently not diagnosed with caries or MIH.

Before the dental examinations began, all examiners (FF, RG, HS, JK, NK) were informed in detail about the study protocol, trained in the clinical procedures, and calibrated. As a result, the reliability for the detection of MIH was documented as Kappa values (intra-investigator reproducibility: 0.484 to 1.000; inter-investigator reproducibility: 0.371 to 0.956) [5]. Therefore, the work group reached at least moderate to excellent agreement on their clinical decisions [34, 35].

### Retrospective questionnaire

All the 8- to 10-year-olds who participated (*N* = 5,418) were given a questionnaire for their parents to fill out and return to the study team after the examination. A prepaid envelope was provided for this purpose. A total of 1,456 questionnaires were returned to the working group. This corresponds to a response rate of 26.9%. Following the digitization of the questionnaires, which was carried out using a customized data entry tool (EpiData, version v4.6.0.6, Denmark), the data sets were checked for completeness and plausibility. Incomplete and obviously inconsistent questionnaires were removed from the data set (*N* = 141). Ultimately, 1,315 data sets containing both clinical data and information from the questionnaire were available for further analysis.

The questionnaire included questions on common socioeconomic determinants of (dental) health as well as detailed documentation options for childhood diseases and medications. Importantly, the family’s socioeconomic status was determined according to the Brandenburg social index [36], which served as a relevant confounder in the statistical data exploration. The calculation was described previously [3], and each individual was dichotomized into low, middle or high socioeconomic status. Furthermore, typical childhood diseases (*N* = 27), including infections with fever, middle ear infections, respiratory diseases such as bronchitis, asthma or lung diseases, dermatologic diseases, and eye diseases, and others, were recorded. Data on childhood diseases were reported in one-year intervals from 0 to 6 years of age and then in one coalesced interval from 7 to 10 years of age. Notably, a medically confirmed diagnosis was required for a positive event. In addition to childhood diseases, operations under general anaesthesia, medications with antibiotics, and painkillers were also queried according to the aforementioned age structure. Owing to the retrospective nature of the study, a differentiated recording of any antibiotic or analgesic medication was not carried out. The questionnaire was extensively pretested and validated [37].

### Statistical analysis

The questionnaires gathered data on the participants’ demographic characteristics, childhood disease history, reported medication use and surgeries under general anaesthesia. The collected data were summarized and tabulated accordingly. The demographic characteristics were used to determine the participants’ socioeconomic status. Based on the clinical examination, participants were assigned to an MIH diagnosis if at least one first permanent molar demonstrated an MIH lesion [8, 33]. Furthermore, the number of MIH-affected teeth was calculated.

A marginalized zero-inflated negative binomial regression model was used for data analysis. This model is particularly suited for handling overdispersed count outcomes with and without zeros. It provides two estimates in its output: adjusted odds ratios (aORs) and incidence rate ratios (IRRs) [38–40]. In this analysis, MIH status (count variable ranging from 0 to 12) was the dependent variable, sex (male or female), age (count variable), and socioeconomic status (count variable ranging from 4 to 10) were set as confounders, and the history of childhood disease was the independent variable. Univariate models were created for each of the reported diseases. To account for participant clustering, robust standard errors were clustered at the administrative district level. Five administrative districts were defined: Upper Bavaria, Middle Franconia, Lower Franconia, Swabia, and others (Lower Bavaria, Upper and Lower Palatinate districts were merged into one due to low population density). The same univariate analysis was repeated for the reported use of analgesics, antibiotics, or prescription medications, as well as operations under general anaesthesia. P-values ≤ 0.05 were considered to indicate statistical significance. The 95% confidence intervals (CIs) are reported. Data were analysed using *Stata* (StataCorp LLC, College Station, TX, USA) and the *mzinb* package.

## Results

The study sample comprised 1,315 participants with a mean (± SD) age of 9.7 years (± 0.6), of whom 697 (53.0%) were male and 618 (47.0%) were female. This cohort exhibited low caries experience, with mean idmft and IDMFT values of 1.0 (± 1.9) and 0.3 (± 0.9), respectively. Regarding MIH, 263 children (20.0%) were diagnosed, with a mean of 3.3 (± 2.0) MIH-affected teeth per child. The mean number of MIH teeth in the entire population was 0.7 (± 1.6).

**Fig. 1:**
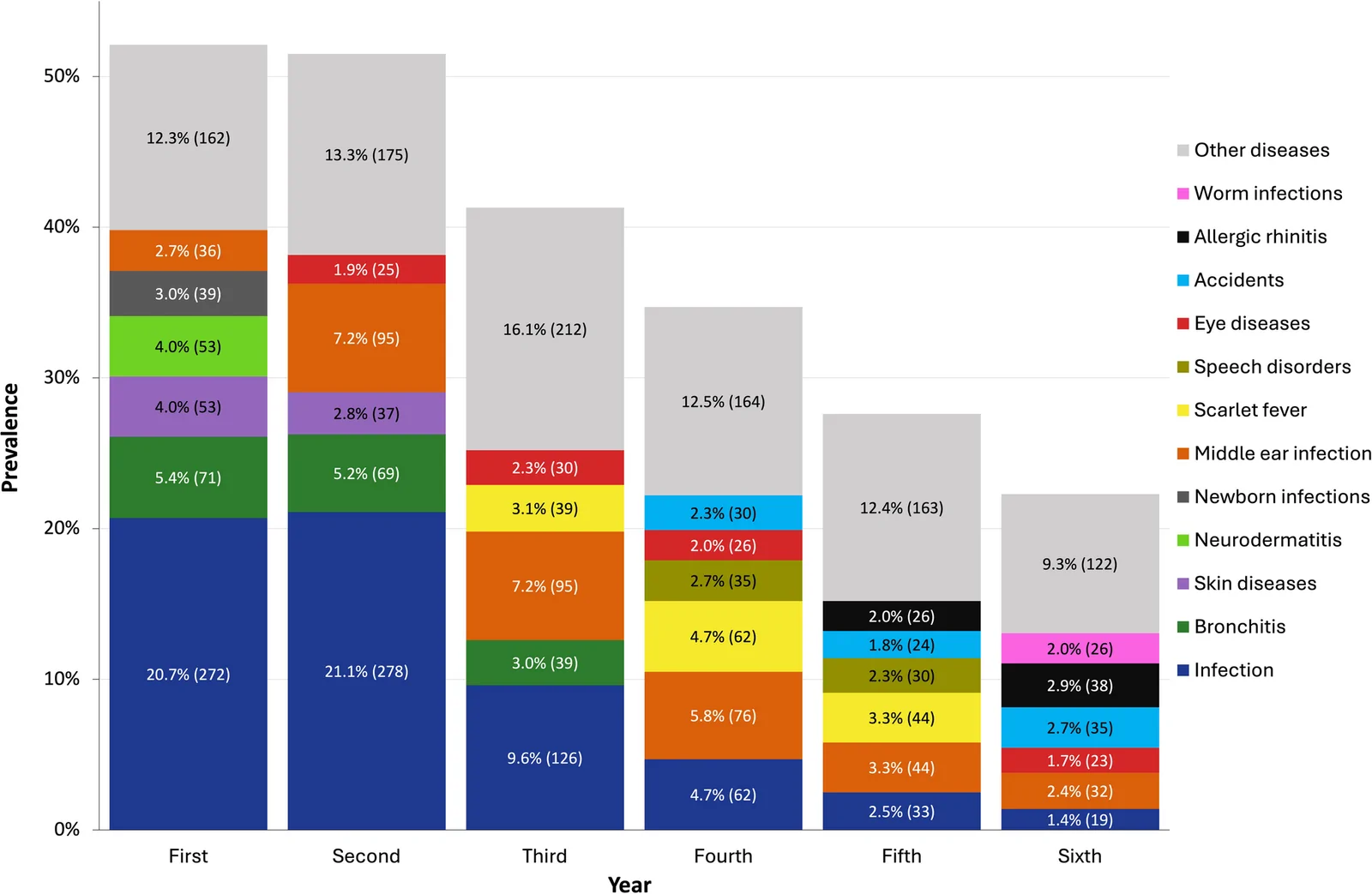
The stacked column chart illustrates the reported percentages and frequencies of childhood diseases for the first 6 years of life in the investigated population of 1,315 Bavarian 8- to 10-year-olds. Multiple diagnoses per individuum were possible

The documented frequencies of early childhood illnesses for the first six years of life are summarized in Fig. 1. Infections with fever were reported most frequently at 20.7% (first year of life) and 21.1% (second year of life) (Fig. 1). Bronchitis was reported at a considerably lower prevalence of 5.4% in the first year of life, and a similar prevalence of 5.2% was documented in the second year of life. The occurrence of skin diseases (4.0%) and neurodermatitis (4.0%) was rarely reported during the first year of life; in the following years, the incidence remained low. In contrast, the reported frequency of middle ear infections increased from the first (2.7%) to the second (7.2%) and third years of life (7.2%). In addition, a large number of childhood diseases were reported, which were recorded with (very) low frequencies.

A descriptive and exploratory analysis of reported early childhood diseases in relation to the presence of MIH is presented in Tables 1 and 2. With reference to the potential influence of the first two years of life on the development of MIH, evaluations of this phase of life were prioritized. Infections with fever (9.0%), bronchitis (2.6%), middle ear infections (2.4%), and neurodermatitis/dermatologic diseases (1.3%/1.1%) were the most frequently reported findings (Table 1). In this group, only middle ear infections in the first year of life (aOR 1.91 with 95% CI 1.30–2.80) and bronchitis (IRR 1.25 with 95% CI 1.07–1.46) in the first or second year of life were significantly associated (Table 2). Further significant associations were found for eye diseases, allergies, and cardiac diseases when these conditions were medically diagnosed during the first year of life (Table 2). Taking the second year into account, significant associations were found for worm infections and speech disorders (Table 2); however, all of these significant findings were based on very low frequency rates.

**Table 1:** The table shows the case distribution of childhood diseases in relation to the presence of MIH for the first year of life, for the first two years of life and for the entire period of investigation for 1,315 8- to 10-year-old Bavarians

N (%)	First year of life				First or second year of life			
	Disease not present		Disease present		Disease not present		Disease present	
	MIH	No MIH	MIH	No MIH	MIH	No MIH	MIH	No MIH
Infections with fever	199 (15.1)	844 (64.2)	64 (4.9)	208 (15.8)	145 (11.0)	620 (47.2)	118 (9.0)	432 (32.9)
Middle ear infections	253 (19.2)	1,026 (78.0)	10 (0.8)	26 (2.0)	231 (17.6)	953 (72.5)	32 (2.4)	99 (7.5)
Accident	263 (20.0)	1,049 (79.8)	0	3 (0.2)	259 (19.7)	1,032 (78.5)	4 (0.3)	20 (1.5)
Bronchitis	244 (18.6)	1,000 (76.1)	19 (1.4)	52 (4.0)	229 (17.4)	946 (71.9)	34 (2.6)	106 (8.1)
Scarlet fever	263 (20.0)	1,048 (79.7)	0	4 (0.3)	259 (19.7)	1,036 (78.8)	4 (0.3)	16 (1.2)
Eye diseases	259 (19.7)	1,043 (79.3)	4 (0.3)	9 (0.7)	252 (19.2)	1,025 (78.0)	11 (0.8)	27 (2.1)
Allergic rhinitis	262 (19.9)	1,051 (79.9)	1 (0.1)	1 (0.1)	261 (19.9)	1,043 (79.9)	9 (0.7)	2 (0.2)
Dermatologic diseases	257 (19.5)	1,005 (76.4)	6 (0.5)	47 (3.6)	248 (18.9)	977 (74.3)	15 (1.1)	75 (5.7)
Worm infections	263 (20.0)	1,052 (80.0)	0	0	262 (19.9)	1,049 (79.8)	1 (0.1)	3 (0.2)
Allergies	254 (19.3)	1,033 (78.6)	9 (0.7)	19 (1.4)	251 (19.1)	1,020 (77.6)	12 (0.9)	32 (2.4)
Speech disorders	262 (19.9)	1,050 (79.9)	1 (0.1)	2 (0.2)	257 (19.5)	1,041 (79.2)	6 (0.5)	11 (0.8)
Neurodermatitis	253 (19.2)	1,009 (76.7)	10 (0.8)	43 (3.3)	246 (18.7)	995 (75.7)	17 (1.3)	57 (4.3)
Lung diseases	259 (19.7)	1,032 (78.5)	4 (0.3)	20 (1.5)	255 (19.4)	1,014 (77.1)	8 (0.6)	38 (2.9)
Smallpox	261 (19.9)	1,046 (79.5)	2 (0.2)	6 (0.5)	259 (19.7)	1,041 (79.2)	4 (0.3)	11 (0.8)
ADHD	263 (20.0)	1,051 (79.9)	0	1 (0.1)	263 (20.0)	1,050 (79.9)	0	2 (0.2)
Hearing disorders	261 (19.9)	1,046 (79.5)	2 (0.2)	6 (0.5)	261 (19.9)	1,042 (79.2)	2 (0.2)	10 (0.8)
Asthma	261 (19.9)	1,049 (79.8)	2 (0.2)	3 (0.2)	260 (19.8)	1,042 (79.2)	3 (0.2)	10 (0.8)
Developmental disorders	257 (19.5)	1,039 (79.0)	6 (0.5)	13 (1.0)	256 (19.5)	1,030 (78.3)	7 (0.5)	22 (1.7)
Obesity	263 (20.0)	1,045 (79.5)	0	7 (0.5)	263 (20.0)	1,043 (79.3)	0	9 (0.7)
Measles	263 (20.0)	1,049 (79.8)	0	3 (0.2)	262 (19.9)	1,044 (79.4)	1 (0.1)	8 (0.6)
Newborn infections	252 (19.2)	1,024 (77.9)	11 (0.8)	28 (2.1)	252 (19.2)	1,024 (77.9)	11 (0.8)	28 (2.1)
Cardiac diseases	256 (19.5)	1,040 (79.1)	7 (0.5)	12 (0.9)	255 (19.4)	1,039 (79.0)	8 (0.6)	13 (1.0)
Lactose intolerance	262 (19.9)	1,048 (79.7)	1 (0.1)	4 (0.3)	261 (19.9)	1,045 (79.5)	2 (0.2)	7 (0.5)
Pertussis	262 (19.9)	1,049 (79.8)	1 (0.1)	3 (0.2)	262 (19.9)	1,045 (79.5)	1 (0.1)	7 (0.5)
Blood diseases	261 (19.9)	1,049 (79.8)	2 (0.2)	3 (0.2)	261 (19.9)	1,048 (79.7)	2 (0.2)	4 (0.3)
Diabetes mellitus	263 (20.0)	1,052 (80.0)	0	0	263 (20.0)	1,051 (79.9)	0	1 (0.1)
Coeliac disease	263 (20.0)	1,052 (80.0)	0	0	263 (20.0)	1,052 (80.0)	0	0

**Table 2:** Marginalized zero-inflated negative binomial regression models examining the incidence rate ratios (IRRs) and adjusted odds ratios (aORs) for the total number of teeth affected by MIH and disease occurrence

	First year of life		First or second year of life	
	IRR (95% CI)	aOR (95% CI)	IRR (95% CI)	aOR (95% CI)
Infections with fever	1.16 (0.88–1.51)	1.38 (0.85–2.23)	1.17 (0.91–1.49)	1.11 (0.87–1.41)
Middle ear infections	1.18 (0.98–1.43)	1.91 (1.30–2.80)	1.18 (0.98–1.43)	1.34 (0.94–1.90)
Accident	-	-	1.16 (0.43–3.10)	0.79 (0.31–2.00)
Bronchitis	1.35 (0.76–2.41)	1.62 (0.67–3.92)	1.25 (1.07–1.46)	1.19 (0.89–1.58)
Scarlet fever	0.83 (0.37–1.89)	1.80 (0.64–5.03)	0.43 (0.21–0.87)	-
Eye diseases	1.52 (1.09–2.12)	1.97 (0.91–4.23)	1.47 (0.80–2.70)	1.49 (0.94–2.38)
Allergic rhinitis	1.79 (0.37–8.79)	-	1.18 (0.73–1.90)	2.40 (0.36–16.06)
Dermatologic diseases	0.65 (0.22–1.89)	0.50 (0.18–1.38)	0.83 (0.40–1.71)	0.66 (0.46–0.94)
Worm infections	-	-	1.33 (1.14–1.55)	0.86 (0.45–1.67)
Allergies	1.40 (1.03–1.91)	2.50 (1.26–4.96)	1.40 (0.90–2.19)	1.98 (1.18–3.32)
Speech disorders	0.94 (0.19–4.52)	3.32 (0.06–198.2)	1.86 (0.90–3.88)	1.77 (1.19–2.61)
Neurodermatitis	0.93 (0.34–2.54)	0.94 (0.43–2.03)	1.12 (0.59–2.13)	1.12 (0.66–1.90)
Lung diseases	1.09 (0.56–2.11)	0.75 (0.54–1.03)	1.14 (0.83–1.58)	1.07 (0.56–2.05)
Smallpox	1.11 (0.14–8.59)	1.01 (0.27–3.78)	0.93 (0.39–2.21)	1.25 (0.66–2.34)
ADHD	-	-	-	-
Hearing disorders	0.50 (0.14–1.83)	3.30 (0.92–11.90)	0.46 (0.18–1.14)	-
Asthma	1.42 (0.58–3.52)	2.67 (0.62–11.52)	0.86 (0.31–2.37)	0.97 (0.26–3.59)
Developmental disorders	1.36 (0.80–2.32)	1.49 (0.45–4.92)	1.23 (0.43–3.47)	0.94 (0.25–3.43)
Obesity	-	-	-	-
Measles	-	-	0.61 (0.20–1.87)	0.33 (0.15–0.74)
Newborn infections	1.08 (0.59–2.00)	1.65 (0.94–2.90)	-	-
Cardiac diseases	2.18 (1.43–3.34)	1.86 (1.55–2.23)	2.26 (1.41–3.65)	1.97 (1.45–2.68)
Lactose intolerance	-	-	-	-
Pertussis	1.13 (0.21–6.12)	1.31 (0.06–26.99)	0.55 (0.08–3.81)	0.49 (0.04–5.94)
Blood diseases	1.88 (0.41–8.58)	1.84 (0.20–16.69)	1.56 (0.35–6.92)	1.35 (0.19–9.51)
Diabetes mellitus	-	-	-	-
Coeliac disease	-	-	-	-

In all the models, the child’s sex, age, and socioeconomic status were included as confounding variables. Participants were clustered in administrative districts.

Bold numbers indicate a statistically significant association (*p* ≤ 0.05). For some diseases, no estimates are reported (–) because the model exhibited convergence issues

**Fig. 2:**
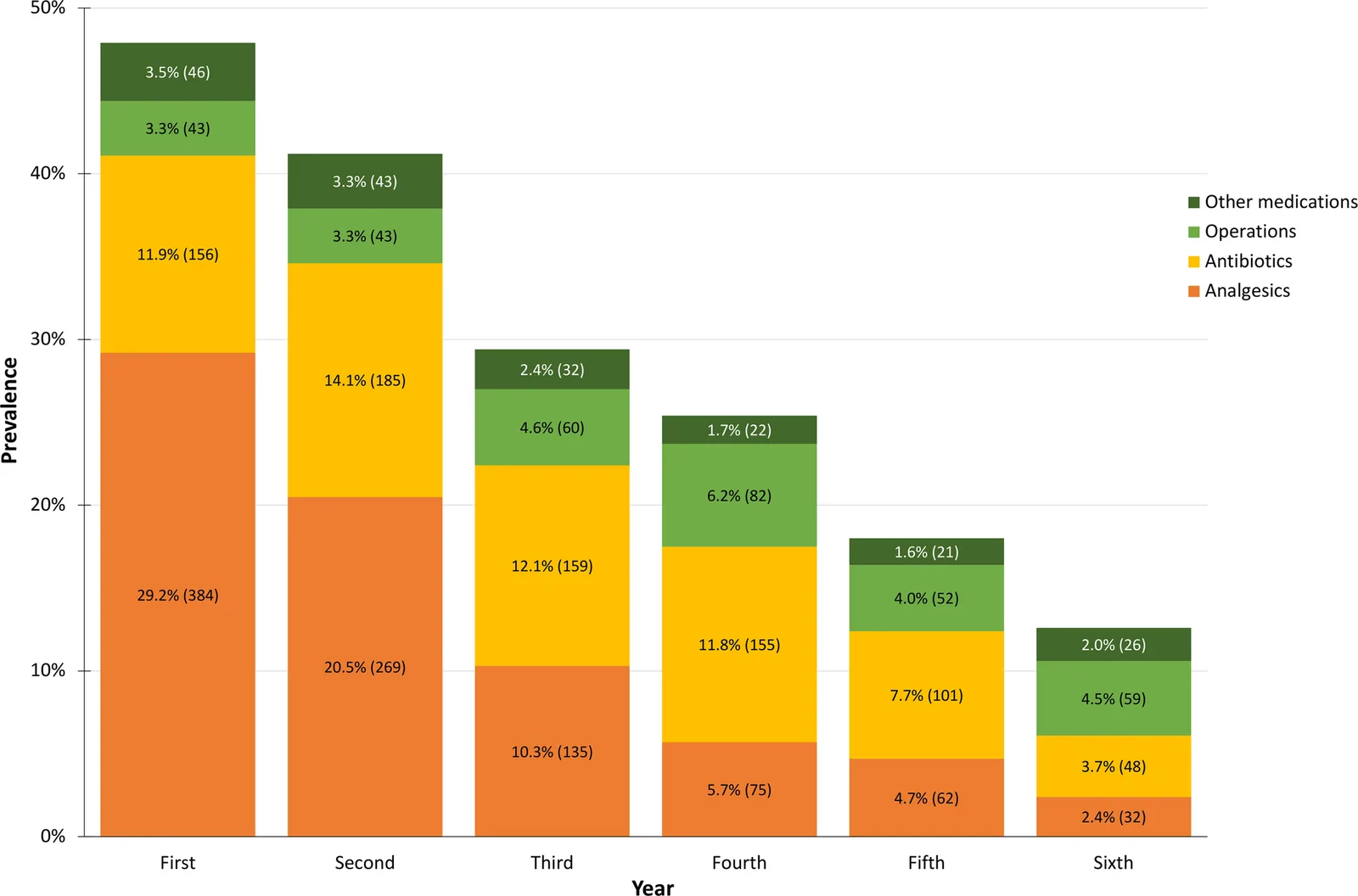
The stacked column chart illustrates the reported percentages and frequencies of analgesics, antibiotics and other medications, as well as operations under general anaesthesia for the first 6 years of life in the investigated population of 1,315 Bavarian 8- to 10-year-olds. Multiple reports per individuum were possible

Based on the available data, it also appears that analgesics were the most common medication in the first two years of life (Fig. 2). A total of 29.2% and 20.5% of all children took analgesics at least once in their first and second years of life, respectively. Retrospectively reported antibiotic use remained largely consistent during the first four years of life. For this age group, between 11.9% and 14.1% of all parents reported that their children had to take antibiotic agents at least once a year (Fig. 2). The results of the regression analyses for medication use and operations under general anaesthesia can be found in Tables 3 and 4. In the case of antibiotic use, consistently significant associations were calculated for both the first (IRR 1.45 with 95% CI 1.15–1.83; aOR 1.86 with 95% CI 1.13–3.05) and the second year of life (IRR 1.35 with 95% CI 1.05–1.77; aOR 1.36 with 95% CI 1.05–1.77).

**Table 3:** The table shows the case distribution for the use of analgesics, antibiotics and other medications, as well as for operations under general anaesthesia (GA) in relation to the presence of MIH for the first year of life, for the first two years of life and for the entire period of investigation for 1,315 8- to 10-year-old Bavarians

N (%)	First year of life				First or second year of life			
	Intervention not used		Intervention used		Intervention not used		Intervention used	
	MIH	No MIH	MIH	No MIH	MIH	No MIH	MIH	No MIH
Analgesics	180 (13.7)	751 (57.1)	83 (6.3)	301 (22.9)	134 (10.2)	528 (40.2)	129 (9.8)	524 (39.9)
Antibiotics	219 (16.7)	940 (71.5)	44 (3.4)	112 (8.5)	180 (13.7)	794 (60.4)	83 (6.3)	258 (19.6)
Other medication	251 (19.1)	1,023 (77.8)	12 (0.9)	29 (2.2)	246 (18.7)	993 (75.5)	17 (1.3)	59 (4.5)
Operations under GA	249 (18.9)	1,023 (77.8)	14 (1.1)	29 (2.2)	241 (18.3)	988 (75.1)	22 (1.7)	64 (4.9)

**Table 4:** Marginalized zero-inflated negative binomial regression models examining the incidence rate ratios (IRRs) and adjusted odds ratios (aORs) for the total number of teeth affected with MIH and the use of analgesics, antibiotics, or other prescription medications, as well as having operations under general anaesthesia (GA)

	First year of life		First or second year of life	
	IRR (95% CI)	aOR (95% CI)	IRR (95% CI)	aOR (95% CI)
Analgesics	1.17 (0.94–1.46)	1.08 (0.86–1.35)	1.08 (0.91–1.27)	0.84 (0.75–0.94)
Antibiotics	1.45 (1.15–1.83)	1.86 (1.13–3.05)	1.35 (1.05–1.77)	1.36 (1.05–1.77)
Other medications	1.75 (0.64–4.80)	1.59 (0.65–3.87)	1.55 (0.95–2.54)	1.10 (0.59–2.08)
Operations under GA	1.63 (0.78–3.39)	1.38 (0.63–3.05)	1.36 (0.82–2.26)	1.04 (0.55–1.97)

In all the models, the child’s sex, age, and socioeconomic status were included as confounding variables. Participants were clustered in administrative districts.

Bold numbers indicate a statistically significant association (*p* ≤ 0.05)

## Discussion

This observational study correlated the prevalence and severity of MIH at the age of 8 to 10 years with the occurrence of multiple childhood diseases, which were retrospectively recorded using a validated questionnaire. The initially formulated null hypothesis had to be accepted in the overwhelming majority of all tested associations. However, in the case of a few children´s diseases, e.g., bronchitis, otitis media, eye diseases, allergies, speech disorders, worm infections and cardiac diseases, a significant association with the presence of MIH was documented (Table 2). The null hypothesis was also confirmed in the case of analgesic intake and surgeries under general anaesthesia. In contrast, antibiotic use in the first and second years of life was associated with significantly increased odds (Table 4); in this case, the null hypothesis formulated at the outset had to be rejected.

Among the most frequently documented childhood diseases in the data set are infections with fever (Fig. 1; Table 1). However, the present data set did not reveal any increased probabilities for the occurrence of MIH (Table 2). These results are consistent with heterogeneous data from various meta-analyses, which, on the one hand, reported significant associations for high fever [6, 7, 10, 41] but, on the other hand, showed no statistical correlations for the occurrence of nonspecific infections [10]. When our own results and the available evidence from meta-analyses are considered, it appears that infections with or without fever do not seem to have a significant influence on the development of MIH. However, treatment of the infection, e.g., with antibiotics, may influence the development of MIH [6].

In this study project, a significant correlation between bronchitis in the first/second year of life and MIH was identified (Table 2). This correlation is generally supported by previously published meta-analyses [6, 7, 10, 42]. However, the low number of cases (Fig. 1; Table 1) suggests that this cannot fully explain the MIH frequency of 20.0%. Since a plausible aetiology is not currently available, the significance of these findings should be interpreted with caution. In connection with respiratory diseases and MIH, asthma is also discussed as a potential cause of MIH. Current meta-analyses confirm this association [6, 10, 41]. However, in the present data set, this association was not significant (Table 2), which is largely consistent with previously published data from the German LISA birth cohort study [43]. However, it should be noted at this point that a conclusive aetiology is also lacking here and that an additional influence of asthma medication must be taken into account [43]. In addition, reference must be made in this context to the low number of asthmatics in the present study population (Table 1), which cannot explain the MIH prevalence of 20.0%. This observation certainly applies equally to eye diseases, allergies, speech disorders, worm infections and cardiac diseases, for which significant associations were documented in the present study project (Table 2), although the frequency of these illnesses can be considered rare (Fig. 1; Table 1). Therefore, significant associations need to be interpreted in relation to the prevalence of the tested variables carefully.

In the case of middle ear infections, the regression model showed a significant adjusted odds ratio of 1.91 (95% CI 1.30–2.80) for the occurrence of MIH (Table 2). Existing meta-analyses have confirmed this association [6, 41]. However, the results from a Finnish observational study [22] are likely to be primarily responsible for these findings. Notably, there is no conclusive explanation as to why otitis media can induce MIH. It is possible that the disease itself is less of an issue here than the potential treatment with antimicrobial substances [6].

In the present data set, a significant association was found between retrospectively reported intake of antibiotics during the first two years of life and the later occurrence of MIH (Table 4). These results are consistent with those of published meta-analyses [6, 7, 44]. In addition, a well-designed animal study [45] demonstrated a plausible aetiology, as the growth of prisms accelerated under the influence of various antibiotics without the enamel prisms mineralizing correctly. As a result, incompletely mineralized tooth enamel was detected [45]. This hypothesis was tested in several observational studies, which revealed that antibiotic administration in the first year of life or in the first three years of life was associated with an increased risk of later MIH development [22, 45]. In addition to the childhood diseases discussed above, the overall frequency of antibiotic use (Table 3) alone cannot explain the 20% prevalence of MIH.

Finally, the methodological strengths and weaknesses of the present study should be discussed. One of the strengths of the study is the relatively large study population of 5,418 clinically examined 8- to 10-year-olds, of whom approximately one-quarter of all parents answered the anonymous questionnaire. This resulted in a total of 1,315 available data sets, which were available for descriptive and exploratory data analysis. With reference to the overall documented participation rate of 50.4% [5] and the questionnaire response rate of 26.9%, it should be noted that the present observational study is not fully representative and can, therefore, be generalized only to a limited extent. A significant limitation of the present study project is the use of a retrospective questionnaire, which was handed over to parents of children aged 8 to 10 years and requested information many from early childhood. The questions about childhood diseases and medications taken were answered by parents or guardians without any medical records. This inevitably leads to recall bias in the information provided and must always be taken into account when the results are interpreted. In addition, the increased MIH prevalence in the present data set of 20% (*N* = 1,315) in contrast to the MIH prevalence of the entire study population (*N* = 5,418) of 17.5% suggests an increased interest from those affected by MIH. Another indicator of positive selection is the documented caries experience in the primary and permanent dentition, which was lower in the present population at 1.0 idmft/0.3 IDMF compared to the total population at 1.6 idmft/0.6 IDMFT [5]. Furthermore, it should be noted that the questionnaire was available only in the German language, which suggests increased participation by parents with German as their native language and excluded families with any other national background. However, this can only be assumed, as data on this aspect could not be collected. Future studies should address such socio-demographic variables precisely as its influence was documented elsewhere [46].

## Conclusion

The results of the present study are consistent with the literature, as mainly no positive associations between the presence of MIH and children’s diseases were found; significant associations were documented for only a few childhood diseases, e.g., bronchitis, otitis media, eye diseases, allergies, speech disorders, worm infections and cardiac diseases. In addition, a significant correlation between antibiotic use and the occurrence of MIH was confirmed. In principle, the few positive correlations were mostly associated with low case numbers, which cannot explain the documented MIH prevalence of 20.0%. Therefore, it can be concluded that (1) childhood diseases and medication intake in the first years of life do not solely explain the occurrence of MIH and that (2) further research is needed to identify the potential reason(s) for MIH.

## Data Availability

The data that support the findings of this study are not publicly available because they contain information that could compromise the privacy of research participants, and participants were not expressly asked about sharing their data for further study. To apply for access to the data, please contact the corresponding author (JK).
